# Comparison Between Ultrasound and Magnetic Resonance Imaging Measurements of the Optic Nerve Sheath Diameter in Patients Undergoing Intracranial Surgery: Prospective Observational Single-Center Study

**DOI:** 10.2196/67480

**Published:** 2026-04-17

**Authors:** Mauricio Giraldo, Luz Maria Lopera, Aly Balbaa, Nelson Gonzalez, Raffael Pereira Cezar Zamper, Michael Mayich, Mel Boulton

**Affiliations:** 1 Department of Anesthesia and Perioperative Medicine, Schulich School of Medicine & Dentistry, Western University, London Health Sciences Centre, 339 Windermere Road, London, ON, N6A 5A5, Canada, 1 519 685 8500 ext 75813; 2 Department of Anesthesia and Perioperative Medicine, Faculty of Medicine, Dalhousie University, Queen Elizabeth II Health Sciences Centre, Halifax, NS, Canada; 3 Department of Family Medicine, Faculty of Medicine, McMaster University, Hamilton Health Sciences, Hamilton, ON, Canada; 4 Department of Neuroradiology, Schulich School of Medicine & Dentistry, Western University, London Health Sciences Centre, London, ON, Canada; 5 Department of Neurosurgery, Schulich School of Medicine & Dentistry, Western University, London Health Sciences Centre, London, ON, Canada

**Keywords:** optic nerve, optic nerve diameter, intracranial pressure, ocular ultrasound, brain tumor

## Abstract

**Background:**

Measuring the optic nerve sheath diameter (ONSD) with ultrasound is a promising, noninvasive way to estimate intracranial pressure (ICP). While magnetic resonance imaging (MRI) provides high-resolution imaging, it is less accessible in urgent or perioperative settings. Comparing ONSD measurements between ultrasound and MRI may help confirm the use of ultrasound in neurosurgical patients.

**Objective:**

The aim of this study is to evaluate how closely ultrasound and MRI measurements of ONSD align in patients undergoing surgery for supratentorial brain tumors.

**Methods:**

This prospective, single-center observational study included 50 adult patients scheduled for elective supratentorial tumor resection. ONSD was measured preoperatively using both transorbital ultrasound and MRI. Measurements were compared using Pearson and Spearman correlation coefficients, the intraclass correlation coefficient, and Bland-Altman analysis.

**Results:**

The average ONSD measured by ultrasound was 5.94 (0.99) mm, compared to 5.75 (SD 1.08) mm via MRI. The two methods showed a strong correlation (Pearson *r*=0.88, *P*<.001) and good agreement (intraclass correlation coefficient=0.86). Bland-Altman analysis showed a mean bias of 0.19 mm (95% limits of agreement: –0.62 to 1.00 mm).

**Conclusions:**

Ultrasound-based ONSD measurements closely matched those obtained by MRI in this patient group. These findings support the use of ultrasound as a practical tool for noninvasive ICP assessment in the perioperative care of patients with intracranial tumors.

## Introduction

Accurate assessment of intracranial pressure (ICP) is essential in the management of patients with a range of neurological conditions [1,2]. Several tools are available for this purpose, including invasive monitoring devices, magnetic resonance imaging (MRI), computed tomography (CT), and ultrasonography (US) [3,4]. While invasive methods remain the gold standard, they carry risks and are not always feasible. MRI and CT offer practical alternatives but can be limited by cost, access, and the need for patient transport, particularly in acute or perioperative settings.

In recent years, ultrasonographic measurement of the optic nerve sheath diameter (ONSD) has gained attention as a practical, noninvasive method for estimating ICP. Ultrasound is widely available at the bedside, relatively inexpensive, and quick to perform. A growing body of evidence supports its use, with studies showing good correlation between ONSD measured by ultrasound and both invasive ICP measurements and imaging-based assessments [5]. This technique has been validated in adult and pediatric populations, including neonates, and across a range of pathologies such as trauma, hydrocephalus, ischemic stroke, and brain tumors. Systematic reviews have also contributed to the evidence base [6,7]. For example, a 2011 meta-analysis by Moretti et al [8] demonstrated the diagnostic accuracy of ONSD US in detecting raised ICP. A 2018 review further emphasized its value, particularly when invasive methods are unavailable or contraindicated [9].

Despite its clinical promise, some uncertainties remain. One ongoing discussion involves the optimal cutoff for diagnosing elevated ICP. While there is no universally accepted threshold, ONSD values of 0.48‐0.50 cm (4.8‐5.0 mm) are commonly used to indicate ICP above 20 cm H_2_O or 20 mm Hg [10-15], with several studies reporting high sensitivity and specificity with these values. For instance, Kimberly et al (2008) [16] reported that an ONSD exceeding 5.0 mm was a strong predictor of raised ICP.

Technique is another area where standardization is still evolving. The typical approach involves measuring the sheath in the transverse plane, 5 mm posterior to the globe [17]. However, anatomical differences between individuals, particularly in the transverse diameter of the eyeball (ETD), may influence interpretation. Some researchers have proposed using the ONSD/ETD ratio to adjust for these variations, with values above 0.19 potentially indicating elevated ICP. While ETD ranges from 21 to 27 mm in healthy individuals, its use in routine clinical decision-making remains under investigation [17]. Recent publications also highlight the growing role of ocular ultrasound in ICP assessment [18,19]. One open-access study noted that bedside ONSD measurement can provide quick, clinically meaningful information during the perioperative period, especially when formal imaging is not immediately available. Their work supports the idea that ultrasound is becoming an increasingly practical addition to routine neurologic monitoring, helping clinicians recognize changes in intracranial dynamics without interrupting patient flow [19].

This study aims to explore the use of ultrasound-based ONSD measurement in a specific neurosurgical population: patients with supratentorial brain tumors. These patients are at risk for increased ICP due to mass effect, edema, or obstruction of cerebrospinal fluid pathways. While previous research has shown a strong correlation between ONSD measured by ultrasound and other modalities, our goal is to assess the agreement between ultrasound- and MRI-derived ONSD values obtained on the same day in a controlled preoperative setting. We also examine the feasibility and accuracy of ultrasound when performed by trained anesthesiologists, a group often involved in perioperative decision-making. Our findings may help clarify the role of ONSD US in situations where access to MRI or CT is limited or delayed.

We conducted a prospective observational study of 50 adult patients undergoing elective supratentorial brain tumor resections at London Health Sciences Center (University Hospital) between July 2021 and May 2023.

## Methods

### Patient Selection

Inclusion criteria were adults (≥18 y) scheduled for elective intracranial surgery involving supratentorial tumors. Patients with previous intraocular lens implantation were included in the study. Patients were excluded if they experienced hemodynamic instability, required emergent or repeat intracranial surgery, or had a history of ocular pathology, including ocular infection, ocular trauma, or prior ocular surgery. All participants provided written informed consent prior to enrollment.

### Ultrasound Examination

On the day of surgery, patients underwent bedside ocular ultrasound prior to anesthetic induction. Ultrasound was performed by a cardiac anesthesiologist formally trained in transthoracic and transesophageal echocardiography and point-of-care ultrasound (POCUS), who had also received a dedicated 2-hour session in standardized ocular ultrasound technique. The session followed current consensus recommendations and included both didactic and hands-on instruction.

Patients were positioned supine with the neck in a neutral position. A high-frequency linear probe (4‐12 MHz, L12-4; Koninklijke Philips NV) was used with sterile water-soluble gel (Aquasonic 100, Parker Laboratories Inc) applied over the closed eyelid, following standard radiologic convention. The ultrasound marker was oriented toward the patient’s right. The optic nerve was visualized in the transverse plane (Figure S1 in Multimedia Appendix 1), and measurements of the ONSD were taken 5 mm posterior to the retina (Figure S2 in Multimedia Appendix 2), perpendicular to the optic nerve axis. Each eye was scanned separately.

Three ONSD measurements were obtained per eye in the transverse plane, and the average of these was used for analysis (Figure S3 in Multimedia Appendix 3). If image quality was suboptimal, additional attempts were made until three acceptable measurements were recorded. All images were stored using a Philips Sparq ultrasound system and Q-Path 5.2.434 software (Telexy Corporation) and anonymized using numeric identifiers per institutional privacy protocols. The ultrasound results were not disclosed to the neuroradiologist performing the MRI measurements.

### MRI Assessment

All patients underwent preoperative brain MRI as part of their standard neurosurgical evaluation. MRI images were obtained using a Siemens Magnetom Vida 3T scanner (Siemens Healthineers). ONSD measurements were performed by a neuroradiologist using standard institutional technique in the axial plane, approximately 5 mm posterior to the globe (Figure S4 in Multimedia Appendix 4). The measurement included the whole optic nerve sheath complex and was obtained at the widest diameter perpendicular to the nerve axis. The MRI results were not disclosed to the anesthesiologist.

### Data Collection

Demographic and clinical variables collected included age, sex, height, weight, and BMI, along with ONSD measurements from both ultrasound and MRI. These covariates were included based on prior literature suggesting that factors such as sex, age, and anthropometric measurements may influence baseline ONSD values or their interpretation in the context of raised ICP. Additionally, clinical characteristics such as tumor size, tumor location, and duration of symptoms were recorded, as these may influence ICP and, by extension, ONSD.

### Statistical Analysis

Data were analyzed using descriptive and inferential statistics. Continuous variables were summarized as means and standard deviations for normally distributed data or medians with interquartile ranges for nonnormal data, while categorical variables were summarized using counts and percentages. Normality of numerical variables was assessed using the Shapiro-Wilk test.

The relationship between ONSD measurements obtained by ultrasound and MRI was evaluated using the Pearson correlation coefficient for normally distributed variables and the Spearman rank correlation when normality assumptions were not met. Differences between ultrasound and MRI ONSD measurements were compared using paired *t* tests for normally distributed data or Wilcoxon signed-rank tests otherwise. Agreement between the two measurement methods was further assessed using the intraclass correlation coefficient (ICC) with a 1-way random-effects model, with significance tested using the *F* test.

A *P* value <.01 was considered statistically significant to account for multiple comparisons and reduce the risk of type I error in this small, exploratory sample. This threshold was defined a priori.

### Ethical Considerations

This study was reviewed and approved by the Research Ethics Board of Western University (protocol number 115045). The study was conducted in accordance with the ethical principles outlined in the Declaration of Helsinki and applicable institutional and national research guidelines. Written informed consent was obtained from all participants prior to enrollment. No compensation was provided to participants.

## Results

### Patient Demographics

A total of 50 patients undergoing brain tumor resection were included in the study, comprising 25 males and 25 females. Participant ages ranged from 21 to 80 years, with a mean age of 55 years (SD 16) and a median of 58 (IQR 21‐80) years. Anthropometric measurements showed a mean weight of 81.7 kg (SD 21.4) and a mean height of 1.71 m (SD 0.11), resulting in a mean BMI of 27.7 (SD 6.0) and a median BMI of 27.0 (IQR 17.3‐42.9). According to World Health Organization BMI classifications, two patients (4%) were underweight, 17 (34%) had normal weight, 19 (38%) were overweight, and 12 (24%) were classified as obese; 5 had type I obesity (BMI 30‐34.9), 5 had type II obesity (BMI 35‐39.9), and 2 had type III obesity (BMI ≥40) (Table S1 in Multimedia Appendix 5).

### ONSD Measurements

The average ONSD measured by ultrasound (US) was 5.94 mm (SD 0.99) in the right eye and 6.15 mm (SD 0.94) in the left eye. In comparison, radiological assessment (RAD) showed slightly higher values of 6.28 mm (SD 0.94) and 6.30 mm (SD 0.86) for the right and left eyes, respectively. Differences between the two methods ranged from –1 mm to 1.8 mm in the right eye and –1 mm to 1.7 mm in the left eye. Statistical analysis suggested a strong positive correlation between US and RAD measurements for both eyes, with correlation coefficients of 0.78 (right eye, Spearman) and 0.84 (left eye, Pearson), both highly significant (*P*<.001). These findings show a good agreement between ultrasound and radiological methods for assessing ONSD in patients undergoing brain tumor resection (Table S2 in Multimedia Appendix 6).

### Correlation Between Imaging Modalities

**Figure 1.**
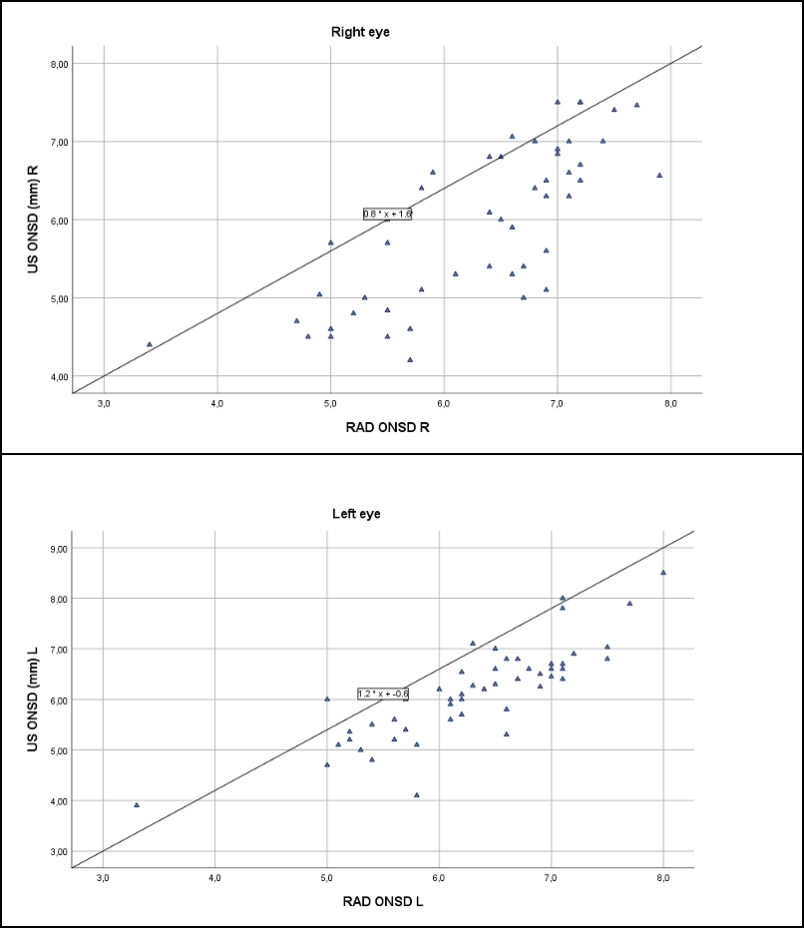
Optic nerve sheath diameter (ONSD) correlation with ultrasound (US) and magnetic resonance imaging (RAD) in patients scheduled for brain tumor resection (University Hospital, July 2021-May 2023, right and left eyes).

There was a clear, statistically significant correlation between ultrasound-measured ONSD values and radiological imaging values. In the right eye, the correlation coefficient was 0.779 (Spearman), and in the left eye, it was 0.836 (Pearson), with both showing *P* values below .001. These findings suggest that the two methods consistently align in their assessment of ONSD, with strong agreement in both eyes (Figure 1).

### Intraclass Correlation Coefficient 

We used ICCs to assess the agreement between ultrasound and radiology measurements of ONSD. For the right eye, the ICC was 0.707 for individual measurements and 0.829 for averaged measurements. The agreement was even stronger in the left eye, with ICCs of 0.823 and 0.903 for single- and average-measures, respectively. All results were statistically significant (*P* values=.001), indicating strong agreement between the two methods. Full details of the ICC values and confidence intervals are available in Table S3 in Multimedia Appendix 7.

## Discussion

This study evaluated the relationship between ONSD measurements obtained via ultrasound and MRI in adult patients undergoing brain tumor resection. We found a strong and statistically significant correlation between the two methods, with minor average differences and high ICCs. These findings suggest that ultrasound may be a practical alternative to MRI for assessing ONSD in the perioperative setting, where rapid, noninvasive evaluation of ICP is often required [20].

In addition to the primary findings, we observed that ultrasound-derived ONSD measurements were generally consistent with MRI-derived measurements, with most values falling within clinically acceptable limits of agreement. Notably, the measurements showed high reproducibility in both eyes, reinforcing the potential utility of ultrasound in routine practice when MRI is unavailable or impractical in patients with supratentorial masses.

Previous research has demonstrated strong associations between increased ONSD and raised ICP [21,22]. Our findings align with those reported by Bäuerle et al [23], who found a correlation between ultrasound- and MRI-based ONSD in a small cohort of healthy volunteers (*r*=0.72). However, their population was younger and nonsurgical. Kerscher et al [24-26] reported even stronger agreement in pediatric patients (*r*=0.976), but their findings are limited to that population. In contrast, our study focuses on adults in a surgical context and provides new evidence supporting the feasibility of ultrasound-assessed ONSD in preoperative neurosurgical care.

A key strength of our approach was the use of POCUS performed by anesthesiologists familiar with the technique. While the clinicians involved had prior ultrasound experience, ONSD-specific training was brief, which reflects the method’s accessibility. Notably, even minimal training has been shown to produce reliable measurements, as seen in studies involving nonphysician trainees [27]. This highlights the practicality of implementing ONSD ultrasound in various clinical settings, especially where MRI is unavailable or delayed.

It is also worth noting that while ONSD ultrasound is most often discussed in the context of trauma or critical care [28], our findings suggest its potential value in elective neurosurgery. In patients with supratentorial tumors, where intraoperative ICP changes can have serious consequences, the ability to assess ONSD in real time could support timely decision-making. Given the growing interest in using POCUS for perioperative neuromonitoring, these findings may help guide future protocols, especially in resource-limited environments.

However, several limitations must be acknowledged. First, the study did not assess interobserver variation, which limits conclusions about the method’s broader applicability to other clinicians or settings. Second, although trained anesthesiologists performed the ultrasound scans, access to formal ultrasound education may not be universal, especially in remote or low-resource centers. Third, our sample included only patients with tumoral disease; thus, the findings may not extend to other intracranial pathologies, such as hemorrhage or infection. Finally, the study’s sample size, while larger than some previous comparisons, remains relatively small and limits the strength of conclusions that can be drawn.

Despite these limitations, this study provides valuable insight into the use of ultrasound for optic nerve evaluation in neurosurgical patients. By demonstrating a strong correlation with MRI, our findings support further exploration of ONSD ultrasound as a screening or monitoring tool for raised ICP, particularly in settings where MRI is unavailable or impractical. Recent open access articles have also pointed out the need for more consistent training and standardized ONSD protocols [32, which aligns with our recommendation for larger, coordinated studies to refine measurement techniques.

Future studies should focus on standardizing training protocols, assessing consistency between different examiners, and evaluating ONSD thresholds across a broader range of intracranial conditions. Larger, multicenter trials could help define normal and pathological ONSD values more precisely and assess how ultrasound-guided ICP screening might impact clinical outcomes. As interest in perioperative neuromonitoring continues to grow, incorporating tools like ONSD ultrasound may help streamline care, improve responsiveness to neurological changes, and broaden access to noninvasive ICP monitoring.

## Supplementary material

10.2196/67480 Multimedia Appendix 1 Optic nerve sheath.

10.2196/67480 Multimedia Appendix 2 Optic nerve measurement 5 mm below retinal layer as indicated in the picture. This is the level to measure the transverse diameter of the optic nerve.

10.2196/67480 Multimedia Appendix 3 Optic nerve diameter measurement.

10.2196/67480 Multimedia Appendix 4 Optic nerve magnetic resonance imaging measurement.

10.2196/67480 Multimedia Appendix 5 Optic nerve diameter correlation with ultrasound and magnetic resonance imaging. University Hospital, July 2021-May 2023.

10.2196/67480 Multimedia Appendix 6 Optic nerve diameter correlation with ultrasound (US) and magnetic resonance imaging (RAD) in patients scheduled for brain tumor resection. University Hospital, July 2021-May 2023.

10.2196/67480 Multimedia Appendix 7 Interclass correlation coefficient.

## Abbreviations

CT: computed tomography
ETD: transverse diameter of the eyeball
ICC: intraclass correlation coefficient
ICP: intracranial pressure
MRI: magnetic resonance imaging
ONSD: optic nerve sheath diameter
POCUS: point-of-care ultrasound
RAD: radiological assessment
US: ultrasonography
