# Supplementary figures and images for: Comparison Between Ultrasound and Magnetic Resonance Imaging Measurements of the Optic Nerve Sheath Diameter in Patients Undergoing Intracranial Surgery: Prospective Observational Single-Center Study
Source: JMIR Perioper Med. 2026 Apr 17;9:e67480. doi: 10.2196/67480 (PMC13089628; doi:10.2196/67480)

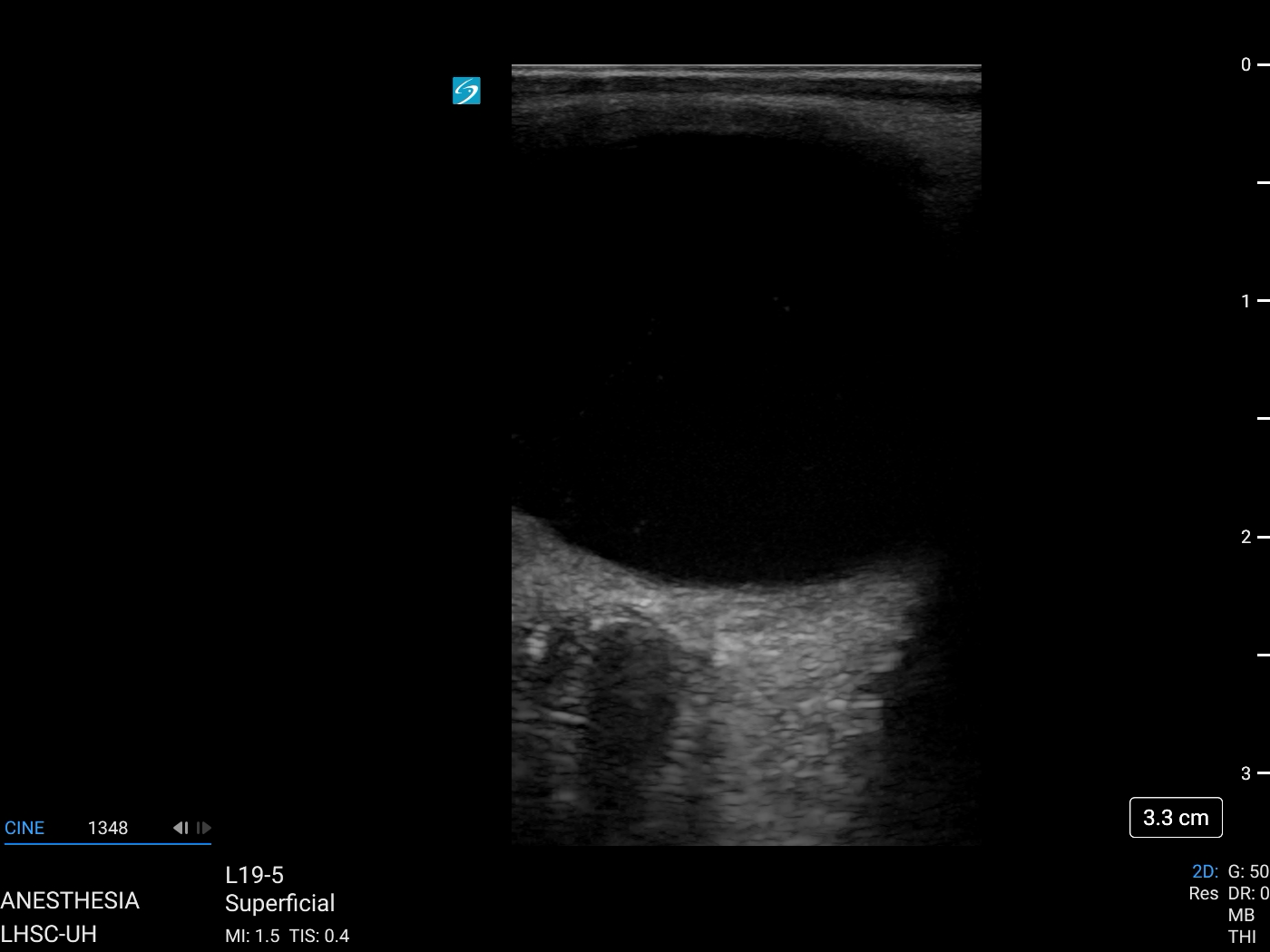
 **Image 1. Optic nerve sheath**

Supplement: Multimedia Appendix 1 [file periop-v9-e67480-s001.docx]

**Image 3. Optic nerve diameter measurement**


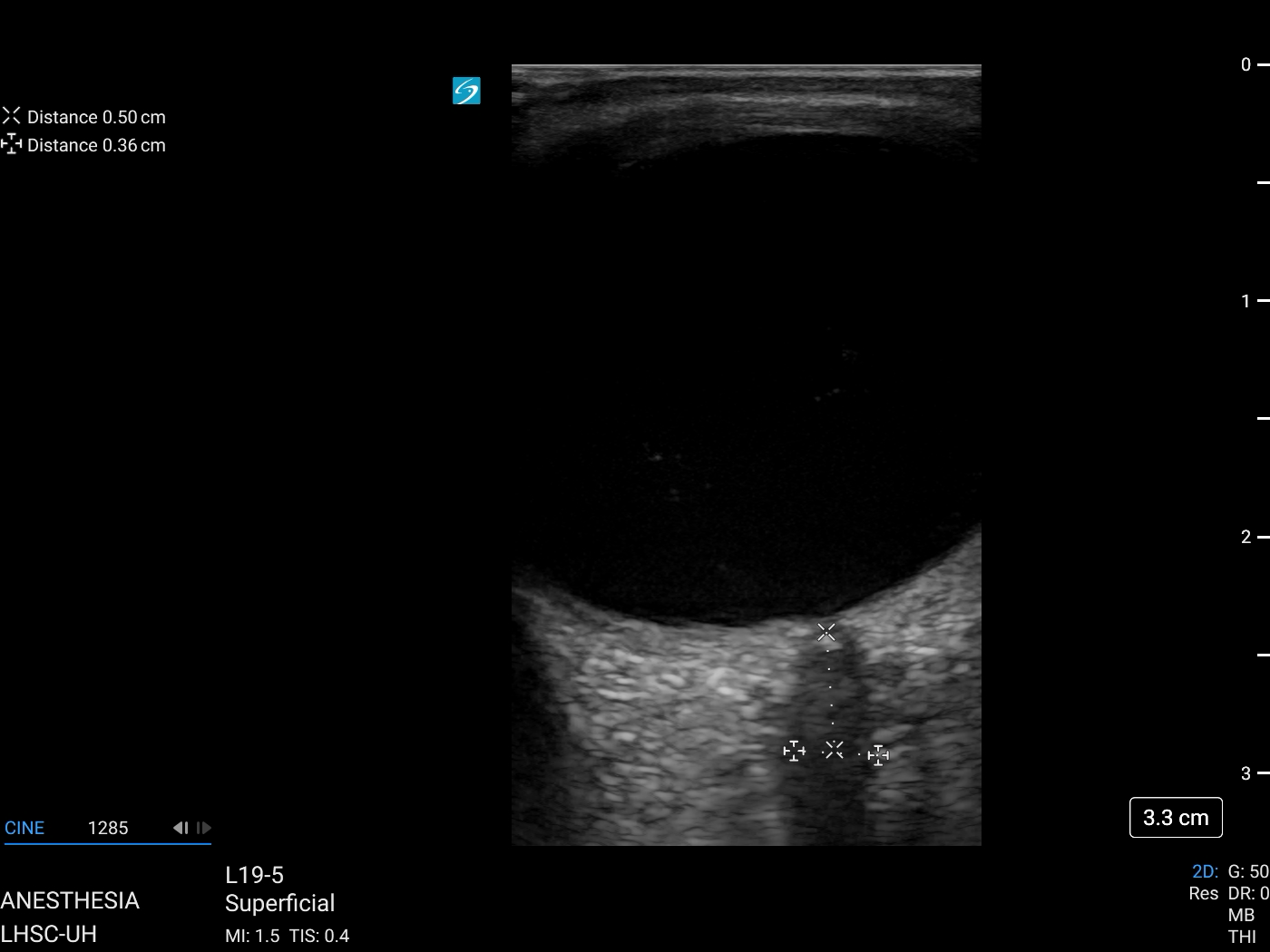

Supplement: Multimedia Appendix 3 [file periop-v9-e67480-s003.docx]

**
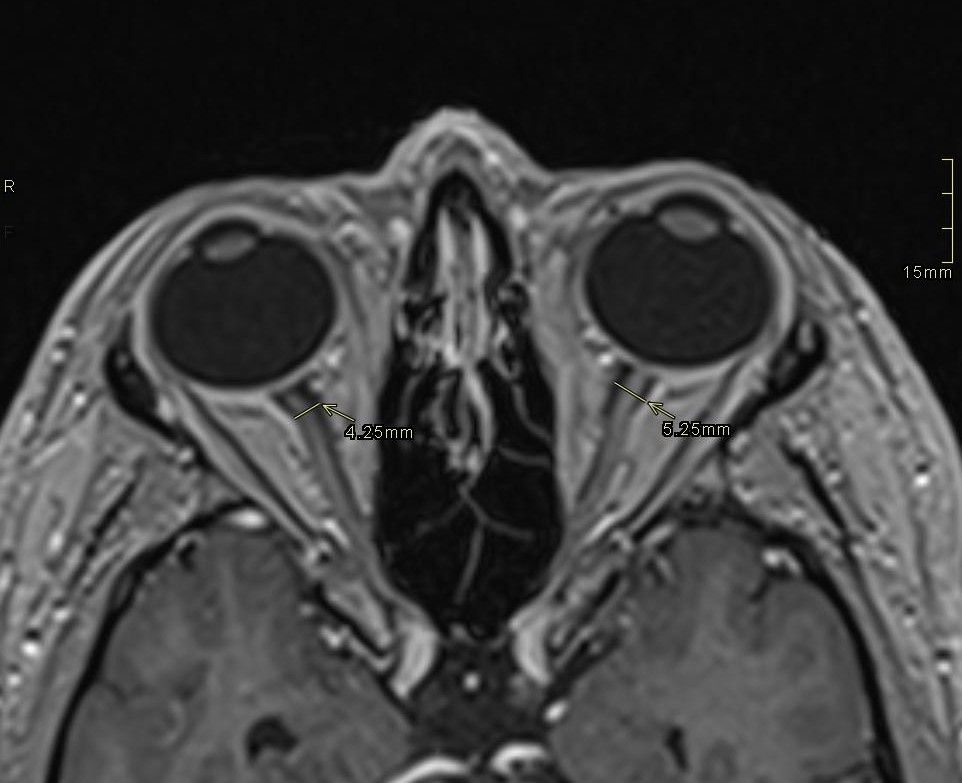
Image 4. Optic nerve MRI measurement.**

Supplement: Multimedia Appendix 4 [file periop-v9-e67480-s004.docx]

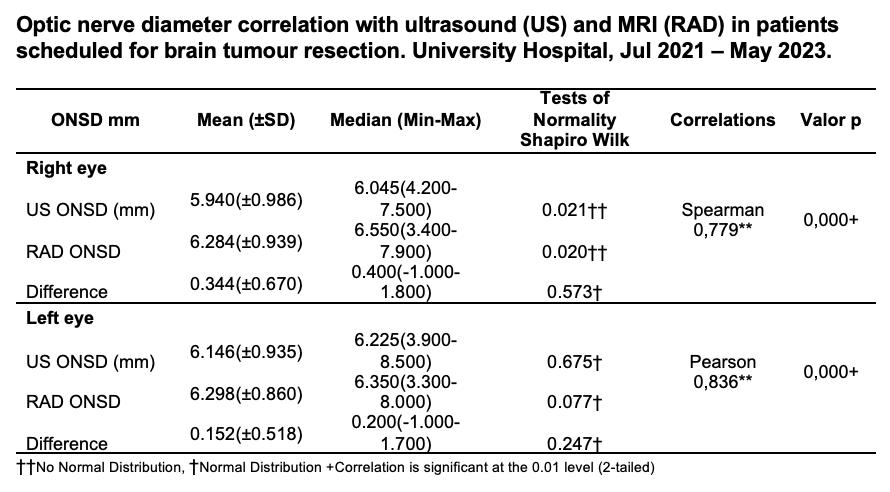

Supplement: Multimedia Appendix 5 [file periop-v9-e67480-s005.png]

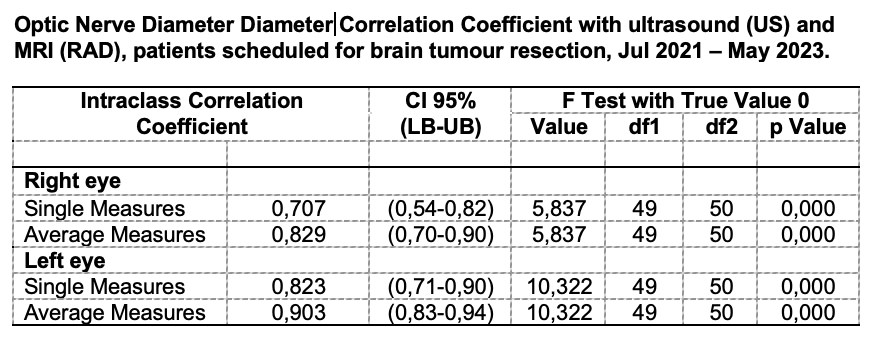

Supplement: Multimedia Appendix 7 [file periop-v9-e67480-s007.png]
