# Supplementary material for: Comparison Between Ultrasound and Magnetic Resonance Imaging Measurements of the Optic Nerve Sheath Diameter in Patients Undergoing Intracranial Surgery: Prospective Observational Single-Center Study
Source: JMIR Perioper Med. 2026 Apr 17;9:e67480. doi: 10.2196/67480 (PMC13089628; doi:10.2196/67480)
Supplement: Multimedia Appendix 2 [file periop-v9-e67480-s002.docx]

**
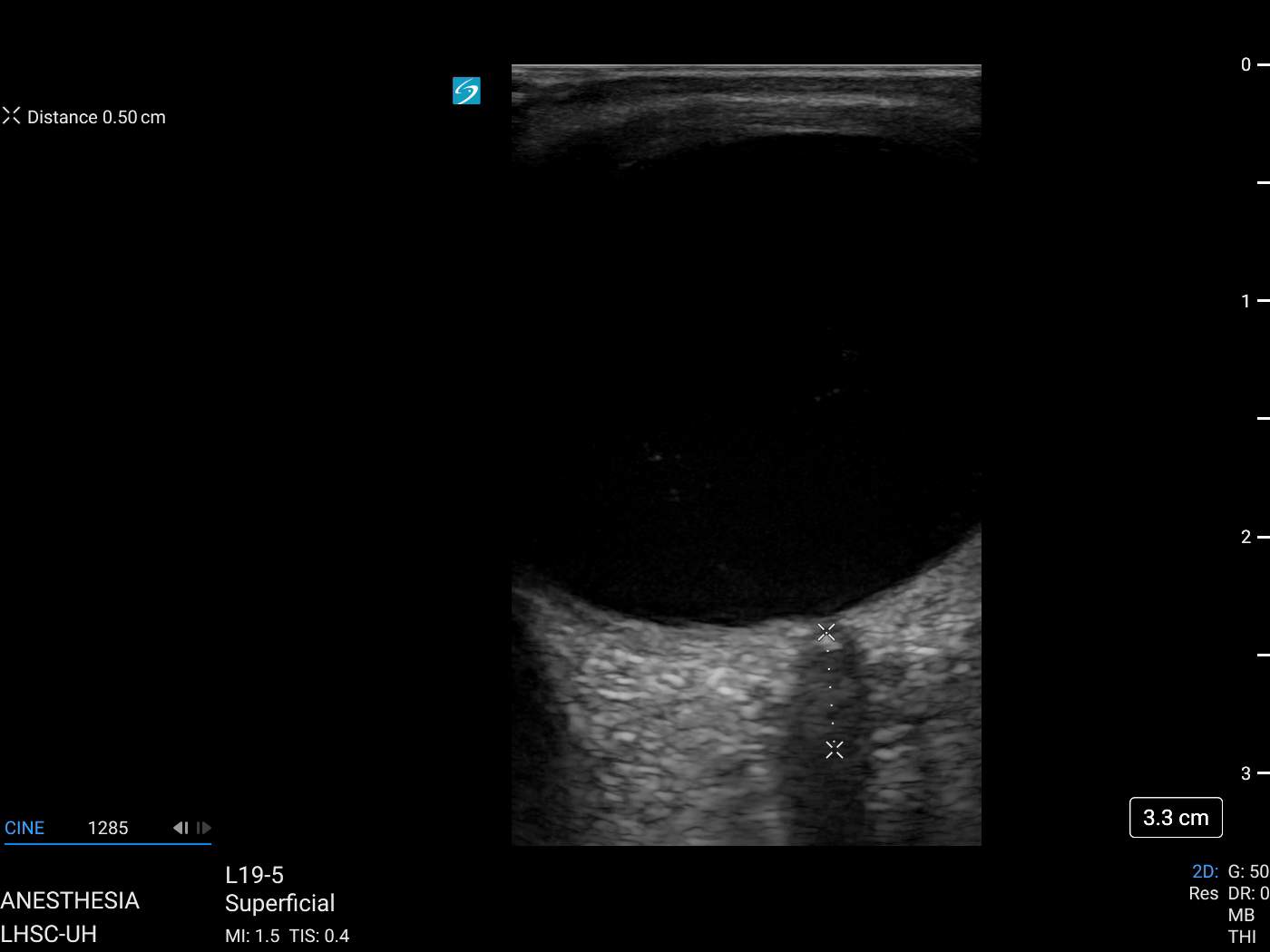
Image 2. Optic nerve measurement 5mm below retinal layer as indicated in the picture. This is the level to measure the transverse diameter of the optic nerve.**
