# Supplementary material for: Comparison Between Ultrasound and Magnetic Resonance Imaging Measurements of the Optic Nerve Sheath Diameter in Patients Undergoing Intracranial Surgery: Prospective Observational Single-Center Study
Source: JMIR Perioper Med. 2026 Apr 17;9:e67480. doi: 10.2196/67480 (PMC13089628; doi:10.2196/67480)
Supplement: Multimedia Appendix 6 [file periop-v9-e67480-s006.docx]

| **ONSD mm** | **Mean (±SD)** | **Median (Min-Max)** | **Tests of Normality Shapiro Wilk** | **Correlations** | **Valor p** |
| --- | --- | --- | --- | --- | --- |
| **Right eye** |  |  |  |  |  |
| US ONSD (mm) | 5.940(±0.986) | 6.045(4.200-7.500) | 0.021†† | Spearman  0,779** | 0,000+ |
| RAD ONSD | 6.284(±0.939) | 6.550(3.400-7.900) | 0.020†† |  |  |
| Difference | 0.344(±0.670) | 0.400(-1.000-1.800) | 0.573† |  |  |
| **Left eye** |  |  |  |  |  |
| US ONSD (mm) | 6.146(±0.935) | 6.225(3.900-8.500) | 0.675† | Pearson  0,836** | 0,000+ |
| RAD ONSD | 6.298(±0.860) | 6.350(3.300-8.000) | 0.077† |  |  |
| Difference | 0.152(±0.518) | 0.200(-1.000-1.700) | 0.247† |  |  |

††No Normal Distribution, †Normal Distribution +Correlation is significant at the 0.01 level (2-tailed)
